# Colistin Induces Oxidative Stress and Apoptotic Cell Death through the Activation of the AhR/CYP1A1 Pathway in PC12 Cells

**DOI:** 10.3390/antiox13070827

**Published:** 2024-07-10

**Authors:** Baofu Xie, Yue Liu, Chunhong Chen, Tony Velkov, Shusheng Tang, Jianzhong Shen, Chongshan Dai

**Affiliations:** 1 National Key Laboratory of Veterinary Public Health and Safety, College of Veterinary Medicine, China Agricultural University, Beijing 100193, China; 2 Department of Pharmacology, Biodiscovery Institute, Monash University Clayton, Melbourne, VIC 3800, Australia

**Keywords:** colistin, oxidative stress, AhR/CYP1A1 pathway, apoptosis, neurotoxicity

## Abstract

Colistin is commonly regarded as the “last-resort” antibiotic for combating life-threatening infections caused by multidrug-resistant (MDR) gram-negative bacteria. Neurotoxicity is a potential adverse event associated with colistin application in clinical settings, yet the exact molecular mechanisms remain unclear. This study examined the detrimental impact of colistin exposure on PC12 cells and the associated molecular mechanisms. Colistin treatment at concentrations of 0–400 μM decreased cell viability and induced apoptotic cell death in both time- and concentration-dependent manners. Exposure to colistin triggered the production of reactive oxygen species (ROS) and caused oxidative stress damage in PC12 cells. N-acetylcysteine (NAC) supplementation partially mitigated the cytotoxic and apoptotic outcomes of colistin. Evidence of mitochondrial dysfunction was observed through the dissipation of membrane potential. Additionally, colistin treatment upregulated the expression of AhR and CYP1A1 mRNAs in PC12 cells. Pharmacological inhibition of AhR (e.g., using α-naphthoflavone) or intervention with the CYP1A1 gene significantly decreased the production of ROS induced by colistin, subsequently lowering caspase activation and cell apoptosis. In conclusion, our findings demonstrate, for the first time, that the activation of the AhR/CYP1A1 pathway contributes partially to colistin-induced oxidative stress and apoptosis, offering insights into the cytotoxic effects of colistin.

## 1. Introduction

Antimicrobial resistance presents a substantial global health challenge that requires immediate attention and concerted efforts to address its repercussions [1]. Polymyxin B or colistin (Polymyxin E) have been employed as final-line drugs to combat infections resulting from multidrug-resistant (MDR) gram-negative bacteria [2,3]. Unfortunately, in clinical practice, polymyxin therapy can cause potential toxic effects, including nephrotoxicity, neurotoxicity, and pulmonary toxicity [4,5]. In some patients, polymyxin therapy-induced neurotoxicity could mainly be manifested in multiple symptoms, such as ataxia, weakness, confusion, apathy, dizziness, vertigo, visual disturbances, and peripheral or orofacial paresthesia [6,7]. In a rat model, colistin methanesulfonate (a prodrug of colistin) treatment at the dose of 150 mg/kg/12 h via intravenous injection induced marked neurotoxicity, which was evident by the abnormal neurobehavioral changes, such as ataxia, muscular weakness, and labored respiration [4]. In addition, our previous studies also found that colistin administration via intravenous injection at the doses of 7.5 or 15 mg/kg/day for seven consecutive days resulted in marked neurobehavioral abnormalities and pathological injury in the brain tissues of mice [8]. Therefore, conducting molecular mechanism research on colistin-induced neurotoxicity is essential for the advancement of protective agents and the development of the next generation of polymyxin drugs.

Currently, the precise molecular mechanisms of colistin-caused neurotoxicity are still not fully characteristically understood. Previous studies showed that colistin treatment could promote the production of excessive reactive oxygen species (ROS), then triggering oxidative stress damage in brain tissues and neuronal cells in vitro [9,10,11,12]. Colistin treatment in neuronal cells may lead to undesired reductions in mitochondrial membrane potential, subsequently activating the mitochondrial apoptotic pathway and ultimately triggering apoptosis in the cells [9,10,11,12]. In addition, several studies also found that colistin-induced neurotoxicity involved multiple signaling pathways, including nerve growth factor (NGF), phosphatidylinositol 3-kinase (PI3K)/protein kinase B (Akt), mammalian target of rapamycin (mTOR), Jun N-terminal kinase (JNK), nuclear factor erythroid 2-related factor (Nrf2), p53, and nuclear factor-κB (NF-κB) pathways [10,12,13,14,15]. Our previous study also found that the activation of autophagy is involved in the process of colistin-induced apoptotic cell death in in vitro cell models (such as N2a, PC12 neuronal cells, and human proximal tubular cells) and plays a protective role [9,10,13,16,17]. Recently, some studies showed that aryl hydrocarbon receptor (AhR) pathway activation contributes to environmental toxins (such as triclosan, Aflatoxin B1, and dibutyl phthalate)-induced neurotoxicity [18,19,20]. AhR could transcriptionally activate the expression of CYP1A1, one of the main cytochrome P450 enzymes, which could release ROS in the form of hydroxyl radicals (OH^•^), superoxide radicals (O_2_^–•^), and hydrogen peroxide (H_2_O_2_) [21]. Recent studies have indicated that pharmacological blockage of the AhR/CYP1A1 pathway has been considered as a promising treatment strategy for various diseases, such as chronic enteritis and neurological and cardiovascular diseases [22]. To date, there is no information on the roles of the AhR pathway in colistin-induced cytotoxicity and neurotoxicity. Therefore, our current study investigated the potential cytotoxic effects of colistin exposure in vitro and the underlying molecular mechanisms of the AhR/CYP1A1 pathway in colistin-caused apoptotic cell death.

## 2. Materials and Methods

### 2.1. Chemicals and Reagents

Sodium fluoride (NaF), α-naphthoflavone (purity ≥ 98%), and sodium orthovanadate (Na_3_VO_4_) were supplied by Aladdin Reagent Company (Shanghai, China). Colistin (sulfate form, 20,195 units/mg) was procured from Sigma (St. Louis, MO, USA). N-acetylcysteine (NAC), phenylmethanesulfonylfluoride (PMSF), bovine serum albumin (BSA), caspases-9 and -3 assay kits, and dimethyl sulfoxide (DMSO) were obtained from Beyotime Biotechnology (Haimen, China). All other reagents utilized in the study met at least analytical grade standards.

### 2.2. Cell Culture

The rat pheochromocytoma cell line (PC12) was derived from the American Type Culture Collection, and the commercial non-differentiated PC12 cell was provided by Shanghai EK-Bioscience Biotechnology Co., Ltd. (Shanghai, China). Cells were kept in the Pharmacology and Toxicology Laboratory at China Agricultural University. Cells were cultured in DMEM with 10% FBS (*v*/*v*) (Gibco, Grand Island, NY, USA) and 1% (*v*/*v*) penicillin-streptomycin (Beyotime Biotechnology, Haimen, China). Cells were maintained in a cell incubator with a standard condition (i.e., at 37 °C and 5% CO_2_). 

### 2.3. Measurement of Cell Viability

Cell viabilities were assessed using a CCK-8 detection kit (Nanjing KeyGEN Biotech Co., Ltd., Nanjing, China), following a previously published method [10]. Briefly, PC12 cells were seeded at 8000 cells per well in a 96-well cell culture plate. The cells were incubated for 24 h before being exposed to varying concentrations of colistin (25, 50, 100, 200, and 400 μM) for an additional 6, 12, and 24 h, after which cell viabilities were determined.

In the examination of oxidative stress’s impact on colistin-induced cytotoxicity, cells were pre-treated with the antioxidant NAC for 2 h before co-treatment with colistin at a final concentration of 400 μM for an additional 24 h. Subsequently, cell viability was assessed. For the investigation of the AhR/CYP1A1 pathway, cells were either transfected with SiCYP1A1 for 24 h or pre-treated with the AhR inhibitor α-naphthoflavone (ANF) at 5 μM (following the methods of a published study [23]) for 2 h before co-treatment with colistin at a final concentration of 400 μM for an additional 24 h. Following this, cell viability was determined. 

### 2.4. Measurement of Apoptosis

Nuclear morphological changes were assessed using the Hoechst 33342 staining method. PC12 cells were exposed to varying concentrations of colistin (100 μM, 200 μM, and 400 μM), either alone or in combination with NAC (2.5 mM) or ANF (5 μM). After 24 h, the cells were stained with Hoechst 33342 solution and then incubated at 37 °C for 30 min. Subsequently, the stained cells were examined and imaged under a fluorescence microscope (Leica Microsystems, Wetzlar, Germany). Cells exhibiting chromatin condensation, nuclear condensation, and the presence of apoptotic bodies were quantified as undergoing apoptosis.

Additionally, cell apoptosis rates were evaluated using a commercial Annexin V-FITC Apoptosis Detection Kit in conjunction with flow cytometry analysis, following the detailed protocol provided by the manufacturer’s instructions (Vazyme Biotech Co., Ltd., Nanjing, China).

### 2.5. Assessment of Oxidative Stress Biomarkers

PC12 cells were exposed to colistin at final concentrations of 100, 200, and 400 μM for 24 h. The oxidative stress biomarkers, such as the activities of catalase (CAT) and superoxide dismutase (SOD) and the levels of malondialdehyde (MDA), were determined using commercial CAT, SOD, and MDA kits (Nanjing Jiancheng, Nanjing, China). Protein concentrations of each sample were determined by using a commercial BCA™ protein assay kit (Beyotime Biotechnology, Haimen, China). 

### 2.6. Measurement of Mitochondrial Membrane Potential (MMP)

Mitochondrial function was evaluated by assessing changes in mitochondrial membrane potential (MMP) using the Rhodamine (Rh) 123 staining method from Beyotime Biotechnology (Haimen, China). Briefly, PC12 cells were exposed to colistin at final concentrations of 100, 200, and 400 μM for 24 h, followed by two washes with PBS (0.01 M, pH 7.2). Subsequently, the cells were incubated with Rh123 staining working solution at 37 °C for 30 min. After a further two washes with PBS (5 min per time), the cells were observed under a fluorescence microscope (Leica Microsystems, Germany).

### 2.7. Levels of Caspases-9 and -3 Activities Measurement

PC12 cells were exposed to colistin at final concentrations of 100, 200, and 400 μM for 24 h, and the activities of caspases-9 and -3 were assessed following the provided protocols of the kits. To investigate the impact of AhR/CYP1A1 on colistin-induced caspase activation, PC12 cells were pre-treated with ANF at a concentration of 5 μM for 2 h, followed by co-treatment with colistin at a final concentration of 400 μM for an additional 24 h. Subsequently, the activities of caspases-9 and -3 were measured.

### 2.8. Gene Knockdown by Short Interfering RNAs (SiRNA)

Small interfering RNAs (siRNAs) for targeting CYP1A1 (Si CYP1A1#1: 5′-GGU CUG AAG AGU CCA CCC GTT-3′ and Si CYP1A1#2: 5′-GAG CUG CUC AGC AUA GUC ATT-3′) were provided (Gene Pharma Company, Shanghai, China). A negative control SiRNA (5′-UUC UCC GAA CGU GUC ACG UTT-3′) was also used. PC12 cells were transfected with the SiRNA using Lipofectamine RNAiMAX reagent (Invitrogen^TM^, ThermoFisher, Waltham, MA, USA) in Opti-MEM™ (Gibco^TM^, ThermoFisher, Waltham, MA, USA) for 24 h. Then, cells were exposed to colistin at the final concentration of 400 μM for an additional 24 h. Finally, the cells were collected for the corresponding analysis. 

### 2.9. Immunofluorescence Staining

Immunofluorescence staining for AhR and CYP1A1 was conducted following a previously published method with minor revisions [10]. Briefly, cells were seeded onto Laser tool holder culture dishes, and after 24 h, they were treated with colistin at a concentration of 400 μM for an additional 24 h. Subsequently, the cells were fixed with 4% paraformaldehyde for 30 min at room temperature. They were then washed twice with PBS (5 min each time), permeabilized with 1% Triton X-100 for 15 min, and treated with a blocking buffer (2% BSA in PBS) for 2 h. The cells were incubated overnight at 4 °C with rabbit anti-AhR (1:200 dilution; ABclonal Technology, Wuhan, China) and CYP1A1 (1:200 dilution; ProteinTech Group, Inc., Chicago, IL, USA) antibodies. Following two PBS washes of 5 min each, the cells were incubated with FITC-labeled goat anti-rabbit IgG secondary antibodies (1:500 dilution; Beyotime Biotechnology, Haimen, China) for 2 h at 37 °C. Finally, the cells were observed using a laser confocal microscope (Leica Microsystems, Germany).

### 2.10. Quantitative RT–PCR

The total RNAs of cell samples were extracted with an RNA Isolation Kit (Vazyme Biotech Co., Ltd., Nanjing, China) and the protocols were followed from a previous study [10]. Detailed information for the primers and protocols is presented in the Supplemental Materials and Methods. 

### 2.11. Western Blot Analysis 

The protein expression in PC12 cells was performed using the Western blot method and the protocols were followed according to the previous descriptions [10]. In brief, the cells, upon treatment, were harvested and lysed with 100 μL of RIPA buffer (Beyotime, Haimen, China) containing a mixture of protease inhibitors (1 mM PMSF, 50 mM NaF, and 5 mM Na_3_VO_4_) for 15 min at 4 °C. Subsequently, the cells were centrifuged at 14,000× *g* for 15 min at 4 °C. The supernatant from each sample was collected post-centrifugation, and protein concentrations were determined. For Western blot analysis, 20 μg of proteins per sample were used. Primary rabbit polyclonal antibody targeting CYP1A1 (1:1000 dilution), as well as mouse monoclonal anti-β-actin (1:1000 dilution), were employed. This was followed by the application of specific anti-mouse or anti-rabbit horseradish peroxidase-conjugated secondary antibodies (1:10,000 dilution; Santa Cruz Biotechnology, Inc., Dallas, TX, USA). Finally, a quantitative analysis of protein expression levels was performed utilizing ImageJ software (V1.8.0.112).

### 2.12. Statistical Analysis

All numerical data were reported as mean ± standard deviation (SD) unless otherwise indicated. Graphs were generated using GraphPad Prism 9.0 software (GraphPad Software, Inc., La Jolla, CA, USA). Statistical analyses were conducted using one-way analysis of variance (ANOVA) with Tukey’s post hoc test for multiple comparisons between groups. A *p*-value below 0.05 was considered statistically significant.

## 3. Results

### 3.1. Colistin Induces Cytotoxicity and Apoptotic Cell Death in PC12 Cells

**Figure 1:**
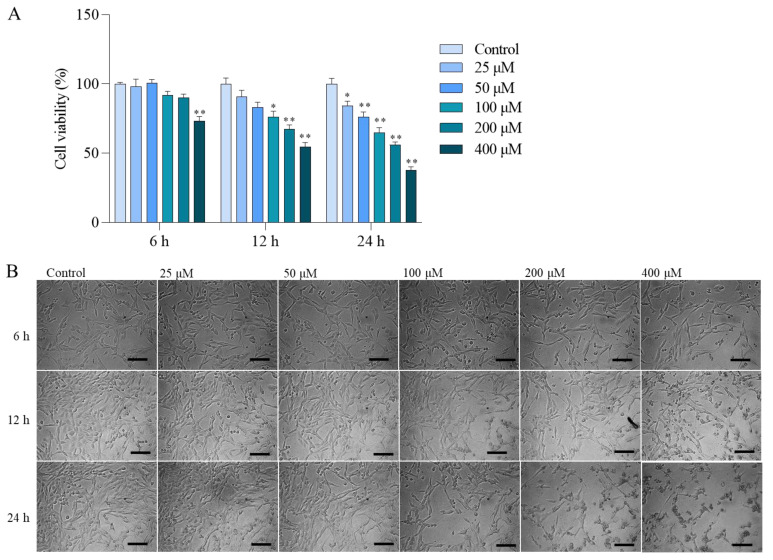
Colistin induces cytotoxicity and morphology changes in PC12 cells. Cells were treated with colistin at concentrations of 25, 50, 100, 200, and 400 μM for 6 h, 12 h, and 24 h. (**A**), the changes in cell viability. Data were shown as mean ± SD (n = 5). (**B**), the changes in cell morphology were observed. The data are presented as mean ± SD (n = 3), with * or ** indicating significance at *p* < 0.05 or 0.01 compared to the untreated control group. In panel (**B**), Bar = 50 μm.

In Figure 1A, colistin treatment in the range of 25–400 μM led to a significant decrease in cell viability in PC12 cells. The cytotoxic effects of colistin were concentration- and time-dependent. After 24 h, treatment with 25, 50, 100, 200, and 400 μM colistin notably reduced cell viabilities to 84.3%, 76.4%, 64.6%, 56.0%, and 37.5% (all *p* < 0.05 or 0.01) compared to untreated cells. Additionally, colistin treatment at 200 μM and 400 μM for 12 and 24 h resulted in decreased cell numbers and morphological alterations such as shrinkage, spindle-like cell bodies, and dendrite fragmentation, as shown in Figure 1B.

**Figure 2:**
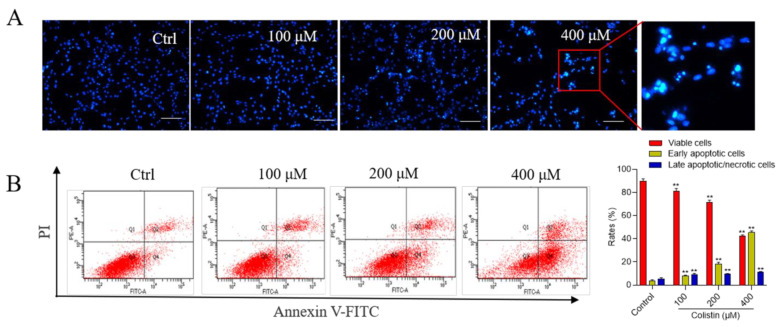
Colistin induces cell nuclear morphology changes and cell apoptosis in PC12 cells. Cells were exposed to colistin at final concentrations of 100, 200, and 400 μM, and after 24 h, nuclear morphology changes and apoptotic rates were assessed. (**A**) The morphological alterations in the cell nucleus using the Hoechst 33342 staining method. Significant nuclear condensation and the presence of apoptotic bodies were identified in the colistin 400 μM treatment group, as evidenced in the magnified image. Bar = 50 μm. (**B**) Cell apoptosis rates and quantitative analysis were conducted using the Annexin V-FITC/PI staining method in combination with flow cytometry analysis. The quadrants represent various cell populations: Q1 for necrotic cells, Q2 for late apoptotic cells, Q3 for live cells, and Q4 for early apoptotic cells. The data are presented as mean ± SD (n = 3), with ** indicating significance at *p* < 0.01 compared to the untreated control group.

Moreover, colistin exposure at final concentrations of 100–400 μM for 24 h induced apoptotic cell death in PC12 cells in a concentration-dependent manner. As illustrated in Figure 2A, treatment with 200 and 400 μM colistin for 24 h led to the appearance of condensed and fragmented chromatin. The apoptotic rates were further evaluated using flow cytometry. As depicted in Figure 2B, compared to the control group, early apoptosis cells increased to 8.1%, 18.2%, and 45.6% (all *p* < 0.01), while late apoptotic/necrotic cells rose to 9.2%, 9.5%, and 11.6% (all *p* < 0.01), respectively.

### 3.2. Colistin Treatment Induces Oxidative Stress in PC12 Cells

**Figure 3:**
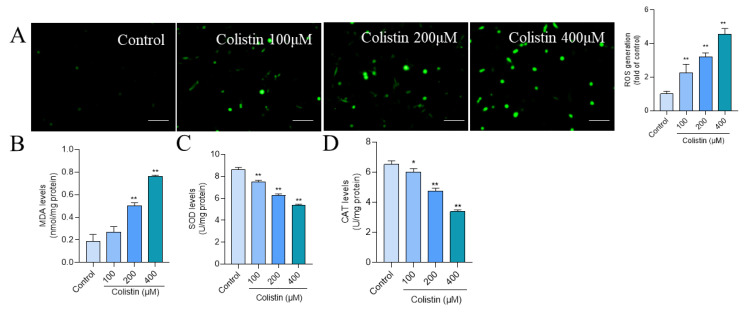
Colistin treatment induces oxidative stress damage in PC12 cells. Cells were treated with colistin at the final concentrations of 100, 200, and 400 μM; after 24 h, the production of ROS and oxidative stress biomarkers were measured. (**A**) The levels of intracellular ROS production. The results of 2,7-dichlorofluorescein diacetate staining (on the left) and the representative images (on the right) are shown (n = 3). Bar = 50 μm. (**B**) The levels of MDA (n = 3). (**C**) The activities of SOD (n = 3). (**D**) The activities of CAT (n = 3). The data are presented as mean ± SD, with * or ** indicating significance at *p* < 0.05 or 0.01, respectively, compared to the untreated control group.

In PC12 cells, treatment with colistin at 100, 200, and 400 μM concentrations for 24 h led to a significant increase in intracellular ROS levels by 2.2-, 3.2-, and 4.5-fold (all *p* < 0.01) (Figure 3A), respectively, compared to the control group. Additionally, levels of oxidative stress biomarkers, such as MDA and the activities of SOD and CAT, were assessed. Notably, exposure to colistin at 400 μM for 24 h resulted in a significant elevation in MDA levels to 0.76 nmol/mg protein (*p* < 0.01) (Figure 3B), accompanied by significant reductions in SOD and CAT activities to 5.35 U/mg protein and 3.38 U/mg protein (both *p* < 0.01) (Figure 3C,D), respectively.

### 3.3. Colistin Treatment Causes Mitochondrial Dysfunction and Caspase Activation in PC12 Cells

**Figure 4:**
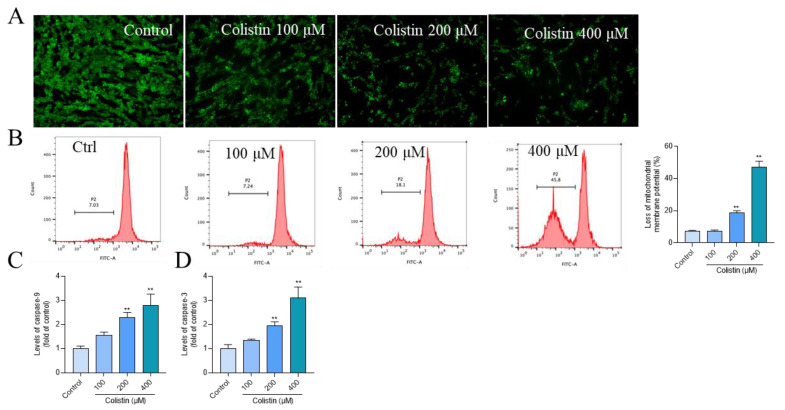
Colistin induces mitochondrial dysfunction and caspase activation in PC12 cells. (**A**), cells were treated with colistin at concentrations of 100, 200, and 400 μM for 24 h; the changes in MMP were measured using Rh123 staining. Bar = 50 μm. (**B**), the corresponding quantitative analysis was carried out using flow cytometry (n = 3). (**C**), the levels of caspase-9 (n = 3). (**D**), the levels of caspase-3 (n = 3). The data are presented as mean ± SD, with ** indicating significance at *p* < 0.01 compared to the untreated control group.

Mitochondrial function was evaluated by assessing MMP using Rh123 staining. Following treatment with colistin at concentrations ranging from 100 to 400 μM for 24 h, a pronounced reduction in green fluorescence (Figure 4A) was observed, indicative of MMP loss. Flow cytometry analysis revealed that the decrease in MMP escalated to 7.7%, 18.6% (*p* < 0.01), and 47.2% (*p* < 0.01) in the 100, 200, and 400 μM colistin treatment groups, respectively (Figure 4B). Colistin treatment upregulated the activities of caspases-9 and -3 (Figure 4C,D). In comparison to the control group, exposure to colistin at 400 μM for 24 h significantly elevated the levels of caspases-9 and -3 by 2.8- and 3.1-fold (both *p* < 0.01), respectively.

### 3.4. NAC Antioxidant Supplementation Attenuates Colistin-Induced Cytotoxicity and Apoptosis in PC12 Cells

**Figure 5:**
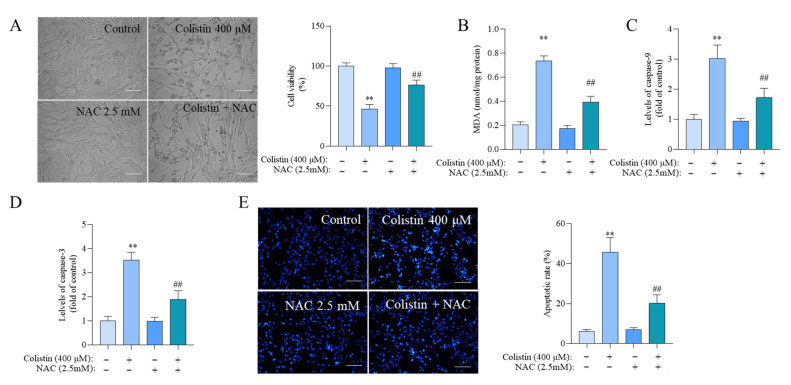
NAC antioxidant supplementation partly reverses colistin-induced cytotoxicity and apoptosis. Cells were treated with either 400 μM colistin or co-treated with 2.5 mM NAC for 24 h. (**A**) Cell morphology changes (**left**) and corresponding viability (**right**) (n = 5) were assessed, respectively. Bar = 50 μm. Additionally, the MDA levels (**B**) and caspase-9 (**C**) and caspase-3 (**D**) activities were measured (n = 3). (**E**), Cell apoptosis rates were determined using the Hoechst 33342 staining method, with representative images on the left and quantitative analysis on the right (n = 3). The data are presented as mean ± SD. ** *p* < 0.01 compared to the control group. ^##^ *p* < 0.01 compared to cells treated with colistin alone.

We further confirmed the roles of oxidative stress in colistin exposure-induced cytotoxicity and apoptosis in PC12 cells. Compared to the colistin alone treatment group, supplementation with antioxidant NAC at a concentration of 2.5 mM significantly attenuated colistin treatment-induced loss in cell viability, increases in MDA levels, caspases activation, and apoptosis (Figure 5). Correspondingly, cell viability increased from 46.6% to 76.5% (*p* < 0.01) (Figure 5A), the levels of MDA decreased from 0.74 nmol/mg protein to 0.39 nmol/ mg protein (*p* < 0.01) (Figure 5B), the levels of caspase-9 activities decreased from 3.0- to 1.7-fold (*p* < 0.01) (Figure 5C), the levels of caspase-3 activities decreased from 3.5- to 1.9-fold (*p* < 0.01) (Figure 5D), and the apoptotic rates decreased from 45.6% to 20.1% (*p* < 0.01) (Figure 5E). NAC treatment did not affect the changes in cell viability, MDA levels, caspase activities, and apoptosis rates (Figure 5).

### 3.5. Colistin Treatment Triggers the Upregulation of AhR and CYP1A1 Proteins in PC12 Cells

**Figure 6:**
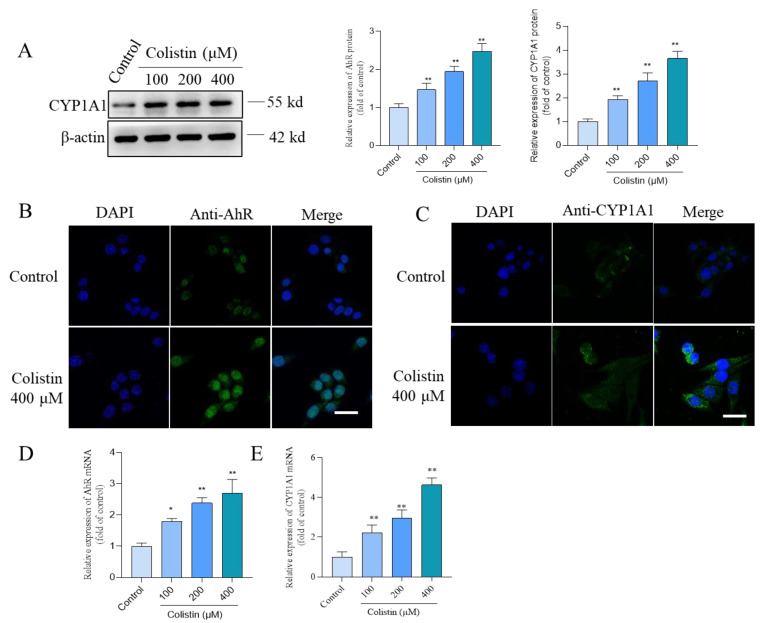
Colistin treatment upregulates the expression of AhR and CYP1A1 proteins and mRNAs in PC12 cells. Cells were treated with colistin at concentrations of 100, 200, and 400 μM for 24 h, and the expression CYP1A1 protein and AhR and CYP1A1 mRNAs were measured. (**A**) the result of CYP1A1 protein expression. The representative images of Western Blot (**left**) and the quantitative analysis (**right**) were shown (n = 3). (**B**) The immunofluorescence staining results of expression and subcellular localization of AhR (**B**) and CYP1A1 (**C**) proteins. Bar = 25 μm. (**D**,**E**), the expression of AhR (**D**) and CYP1A1 (**E**) mRNAs were analyzed using the qRT-PCR method (n = 3). The data are presented as mean ± SD, with * or ** indicating significance at *p* < 0.05 or 0.01 compared to the untreated control group.

Compared to the control group, PC12 cells treated with colistin demonstrated a significant increase in the expression of CYP1A1 proteins and the expression of AhR and CYP1A1 mRNAs. In Figure 6A, treatment with colistin at concentrations of 100, 200, and 400 μM markedly increased CYP1A1 protein expression by 1.9-, 2.7-, and 3.7-fold (all *p* < 0.01). Moreover, immunofluorescence staining indicated that colistin treatment at 400 μM enhanced the expression of nuclear AhR and CYP1A1 proteins compared to untreated cells (Figure 6B,C). Conversely, CYP1A1 expression was localized in the cytoplasm. The expression of AhR and CYP1A1 mRNAs was also evaluated, revealing a significant upregulation upon exposure of PC12 cells to colistin. As depicted in Figure 6D,E, treatment with 400 μM colistin for 24 h substantially increased AhR and CYP1A1 mRNA expression 2.7- and 4.6-fold (both *p* < 0.01), respectively, compared to untreated cells.

### 3.6. Pharmacological Intervention of CYP1A1 Attenuates Colistin Treatment-Induced Loss of Cell Viability, the Production of ROS, Caspase Activation, and Apoptosis

**Figure 7:**
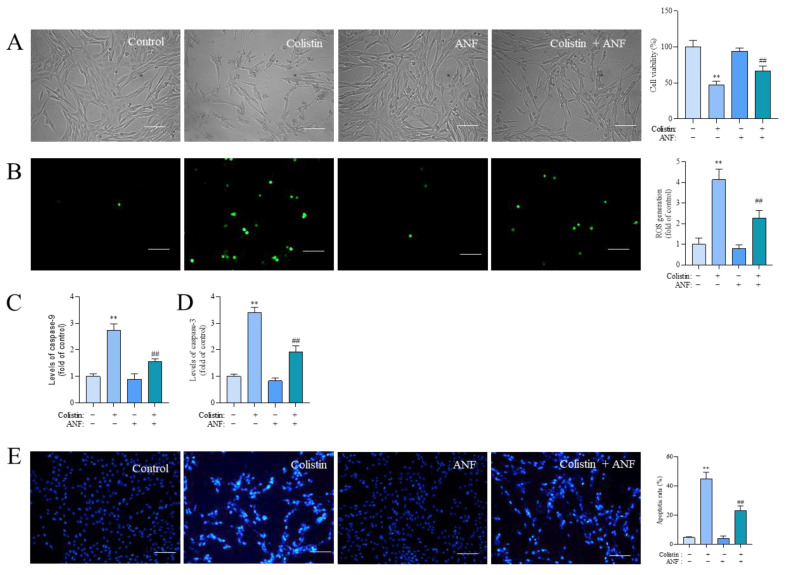
α-naphthoflavone supplementation attenuates colistin-induced cytotoxicity, the production of ROS, caspase activation, and apoptosis. Cells were either treated with 400 μM colistin or co-treated with α-naphthoflavone (ANF) at 5 μM for 24 h. (**A**) The representative images of cell morphology (**left**) and corresponding cell viability (**right**) (n = 5). (**B**) The assessment of intracellular ROS production, presented through 2,7-dichlorofluorescein diacetate staining results (on the **left**) and corresponding quantitative analysis (on the **right**) (n = 3). Bar = 50 μm. (**C**,**D**) The levels of caspases-9 (**C**) and -3 (**D**) activities (n = 3). (**E**) Cell apoptosis rates were determined using the Hoechst 33342 staining method, with representative images on the left and quantitative analysis on the right (n = 3). Bar = 50 μm. The data were reported as mean ± SD. ** *p* < 0.01 compared to the control group. ^##^ *p* < 0.01 compared to cells treated with colistin alone.

Compared to the control group, treatment with 400 μM of colistin significantly induced a decrease in cell viability, promoted ROS production, upregulated caspases-9 and -3 levels, and ultimately triggered cell apoptosis. These adverse effects could be partially ameliorated through co-treatment with ANF. As demonstrated in Figure 7, ANF treatment at 5 μM markedly enhanced cell viability to 67.3% (*p* < 0.01) (Figure 7A), reduced ROS production by 2.3-fold (*p* < 0.01) (Figure 7B), decreased caspase-9 and -3 levels by 1.6-fold and 1.9-fold (Figure 7C,D), and lowered apoptotic rates to 23.3% (*p* < 0.01) (Figure 7E) compared to those in the colistin-alone treatment group. Treatment with ANF alone at a final concentration of 5 μM did not alter cell viability, ROS production, caspases-9 and -3 levels, or apoptotic rates compared to the untreated cells.

### 3.7. CYP1A1 Knockdown Attenuates Colistin-Induced Cytotoxicity, the Production of ROS, and Apoptosis

**Figure 8:**
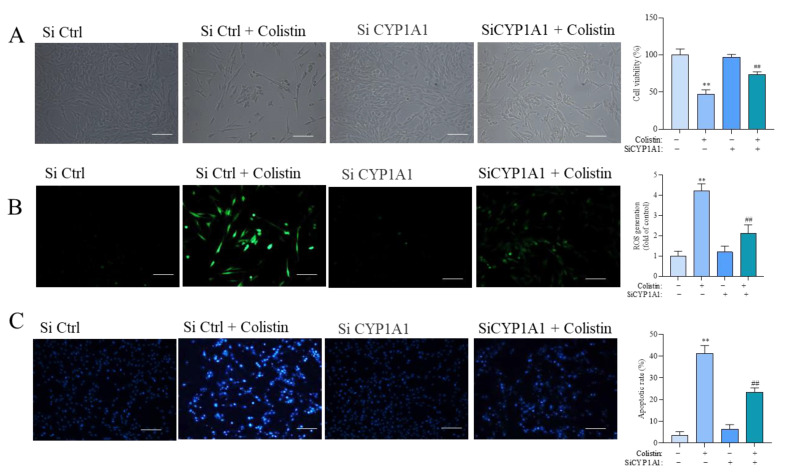
CYP1A1 knockdown reduces colistin-induced cytotoxicity, the production of ROS, and apoptosis. Cells were transfected with SiCYP1A1#1 for 24 h, followed by treatment with 400 μM colistin for an additional 24 h. (**A**) The representative images of cell morphology (on the **left**; Bar = 50 μm) and corresponding cell viability (on the **right**) (n = 5). (**B**) The levels of intracellular ROS production, with the results of 2,7-dichlorofluorescein diacetate staining (on the **left**) and corresponding quantitative analysis (on the **right**) (n = 3). Bar = 50 μm. (**C**) Cell apoptosis rates were assessed using the Hoechst 33342 staining method, with representative images (on the **left**; Bar = 50 μm) and quantitative analysis (on the **right**) conducted (n = 3). ** *p* < 0.01 compared to the control group. ^##^ *p* < 0.01 compared to the cells treated with colistin alone.

We further confirmed the role of CYP1A1 in colistin-caused cytotoxicity and apoptosis in PC12 cells. The expression of CYP1A1 mRNA in PC12 cells transfected with SiCYP1A1#1 and #2 was significantly decreased (Supplementary Figure S1). Then, we found that CYP1A1 knockdown significantly attenuated colistin treatment-induced loss of cell viability, which was increased to 73.4% (*p* < 0.01; Figure 8A). Meanwhile, CYP1A1 knockdown by SiCYP1A1 also significantly reduced colistin treatment-induced production of ROS and apoptosis (Figure 8B,C).

## 4. Discussion

In recent decades, colistin has become a prevailing choice as a final recourse in treating life-threatening infections caused by MDR-negative bacteria globally [24,25]. However, in clinical practices, colistin therapy could cause potentially unwanted adverse effects in patients, such as neurotoxicity, nephrotoxicity, pulmonary toxicity, and skin hyperpigmentation [5,26,27,28]. Understanding the molecular mechanisms of colistin-induced neurotoxicity is crucial for its prevention and effective intervention. The previous studies from animals and in vitro cell models indicated that colistin-caused neurotoxicity is associated with oxidative stress, apoptosis, mitochondrial dysfunction, autophagy, and neuroinflammation [9,11,12,13,15]. However, to date, the precise molecular mechanisms remain unclear, and effective neuroprotective agents are scarce. Therefore, exploring the molecular mechanisms underlying the neurotoxicity of colistin is still indispensable for the development of neuroprotective agents.

The current study revealed that treatment with colistin in the dosage range of 25–400 μM led to a concentration- and time-dependent decrease in cell viability (Figure 1). This is consistent with several previous studies [10,12,13,29]. Zhang et al. found that colistin sulfate treatment-induced cytotoxicity is time-dependent in PC12 cells, and the cell viability was decreased to ~75% when cells were treated with colistin at the final concentration of 125 μg/mL (i.e., it is equal to ~100 μM colistin) for 24 h [29]. In another study, it was reported that colistin treatment at a concentration range of 3.13–1600 μM for 6–48 h could induce cell death in N2a neuronal cells in both concentration- and time-dependent manners [12]. Furthermore, our results found that colistin-induced cytotoxicity in PC12 cells involved oxidative stress, mitochondrial dysfunction, apoptosis, and the activation of caspases and the AhR/CYP1A1 pathway (Figure 2, Figure 3, Figure 4, Figure 5, Figure 6, Figure 7 and Figure 8). 

Previous studies have illustrated that colistin treatment could concentration-dependently induce apoptotic cell death in PC12 or N2a neuronal cells [12,15,29]. In line with these previous findings, current data from Figure 2 show that colistin treatment at concentrations of 100–400 μM could also induce cell apoptosis in PC12 cells. These findings confirm that apoptosis plays a crucial role in the cytotoxicity and neurotoxicity induced by colistin. It is well known that excessive production of ROS can attack lipids, proteins, DNA, and subcellular organelles, ultimately triggering cell apoptosis [30]. In the present study, our data from Figure 3 demonstrate that colistin treatment could induce the excessive production of ROS. Additionally, a notable increase in levels of malondialdehyde (MDA), a biomarker of lipid peroxidation, has been observed [31]. Moreover, the activities of the antioxidant enzymes superoxide dismutase (SOD) and catalase (CAT) were significantly reduced in these cells. SOD facilitates the conversion of O_2_^–•^ into oxygen and H_2_O_2_, which is subsequently processed by CAT into non-toxic H_2_O and oxygen (O_2_) [32]. Prior research has shown that colistin treatment elevates ROS production, lowers SOD and CAT levels, and leads to oxidative stress in N2a and PC12 cells [10,12,29]. Edrees et al. reported that colistin exposure significantly increased MDA levels and decreased CAT activities and reduced glutathione (GSH) levels in rat brain tissues [33]. These findings suggest a crucial role of oxidative stress in colistin-induced cytotoxicity and neurotoxicity.

Mitochondria not only contribute to ROS production but also serve as a primary target. Previous studies have shown that colistin treatment could induce mitochondrial dysfunction in N2a neuronal cells [9,12]. We previously also reported that intravenous administration of colistin at the dose of 15 mg/kg per day for seven days could induce mitochondrial dysfunction in brain tissues [8]. In the current study, we observed that exposing cells to colistin at concentrations of 100–400 μM for 24 h led to a significant loss of mitochondrial membrane potential (MMP), as depicted in Figure 4A,B, which indicates mitochondrial dysfunction. Furthermore, we detected a notable increase in the activities of caspases-9 and -3 in colistin-treated cells, as shown in Figure 4C. Recent research has demonstrated that colistin treatment upregulates the Bax/Bcl-2 ratio, promoting the release of cytochrome C (CytC) from mitochondria, consequently activating caspases-9 and -3, leading to cell apoptosis [9,12]. Edrees et al. reported a significant increase in levels of CytC and caspase-3 proteins in the brain tissues of rats following colistin exposure [33]. Consistent with these findings, our current data show that antioxidant N-acetylcysteine (NAC) supplementation significantly reverses colistin-induced cytotoxicity, caspase activation, and apoptosis in PC12 cells, as illustrated in Figure 5. These findings indicate the involvement of the mitochondrial apoptotic pathway in colistin-induced apoptosis and neurotoxicity, which is partly dependent on ROS production. 

It is reported that colistin exposure could induce increased ROS production in human proximal tubular epithelial (HK2) cells and human A549 cells via the activation of the transforming growth factor-β1 (TGF-β1)/NADPH oxidase 4 (NOX4) pathway [34]. It is reported that NOX4 activation could induce the production of ROS, i.e., O_2_^–•^ [35]. Targeted inhibition of NOX4 can limit the ROS production induced by colistin, thereby significantly reducing mitochondrial dysfunction and apoptosis in cells [34]. Recent studies found that AhR-mediated CYP1A1 overexpression is required in several environmental compounds (i.e., benzo[a]pyrene, polychlorinated diphenyl sulfides, 2,3,7,8-tetrachloro-dibenzo-p-dioxin, and decabromodiphenyl ether) to induce the production of ROS and oxidative stress damage [36,37,38]. CYP1A1 is one of the important downstream genes involved in AhR transcriptional regulation in response to exogenous toxic compounds [36,37,38]. Our current data also show that colistin treatment significantly upregulated the expression of AhR and CYP1A1 proteins and mRNAs (Figure 6). Nuclear AhR expression was significantly increased in colistin-treated PC12 cells (Figure 6). In addition, our results showed that ANF or gene interference of CYP1A1 significantly inhibited colistin-induced cytotoxicity, the production of ROS, caspase activation, and apoptosis (Figure 7 and Figure 8). Similarly, Liu et al. found that knockdown AhR or CYP1A1 by SiRNA could both significantly inhibit polychlorinated diphenyl sulfides-induced production of ROS and toxic effects in HepG2 cells [38]. A recent study found that intraperitoneally injected ANF at the dose of 30 mg/kg/day for 3 days could effectively reduce colchicine exposure-induced acute liver injury via the inhibition of CYP1A1 activities [39]. In a mouse model, ANF supplementation could significantly inhibit the expression of AhR and CYP1A1 proteins, improving high-fat diet-induced liver injury [23]. In another study, it was reported that ANF supplementation could significantly ameliorate H_2_O_2_ exposure-caused SH-SY5Y neuronal cell damage via the inhibition of oxidative stress damage [40]. Taken together, these data suggest that the activation of the AhR/CYP1A1 pathway contributes to colistin-induced cytotoxicity and apoptosis via the induction of ROS. 

There were several limitations in the present study. In line with several previous studies [13,15,29,41,42,43,44], the PC12 cells used in the present study were in the non-differentiated state, which may not mimic neurons to study neurotoxicity. Our current research also lacked confirmation of the primary neuronal cell models and animal models. The previous studies also reported that ANF has a complex pharmacology activity; it is a competitive binding inhibitor for CYP1A1, CYP1A2, CYP1B1, and AhR [45,46,47]. It is unclear whether other CYP1 enzymes are involved in regulating colistin-induced oxidative stress and apoptosis. This is another limitation of our current study. In addition, AhR could also regulate the expression of cyclic AMP response element (CRE)-binding protein (CREB) and Nrf2 proteins, which are two critical survival factors during colistin-induced neuronal cell death [10,11,48]. The precise molecular mechanism remains to be further explored.

## 5. Conclusions

**Figure 9:**
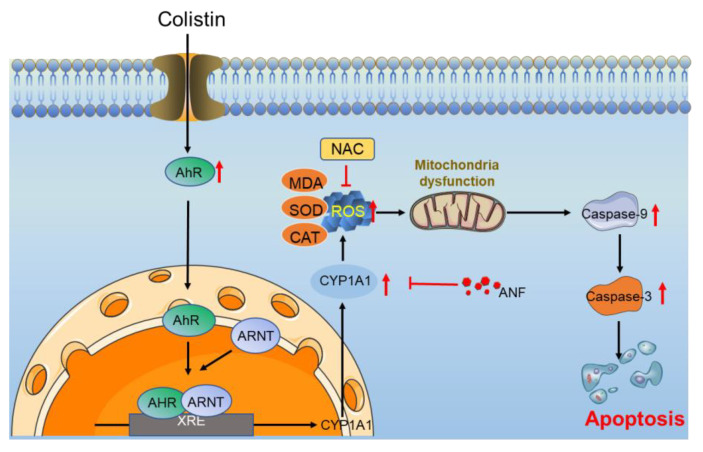
A proposed model of colistin treatment-induced cell apoptosis in PC12 cells. Colistin treatment upregulates the expression of AhR, leading to its nuclear translocation, which subsequently induces the transcriptional expression of CYP1A1, triggers ROS production, and ultimately results in oxidative stress. The excessive ROS levels can further lead to mitochondrial dysfunction, subsequently activating caspases-9 and -3, ultimately resulting in cell apoptosis in PC12 cells. ↑, indicates upregulation.

In conclusion, this current study, for the first time, reveals that colistin-induced cell apoptosis involves the activation of the AhR/ CYP1A1 pathway. A concise working mode diagram of AhR/CYP1A1 pathway-mediated colistin-induced apoptosis is shown in Figure 9. The upregulation of CYP1A1 could partially induce ROS production, cascading into the induction of oxidative stress damage and mitochondrial dysfunction, finally causing cell apoptosis. The pharmacological or genetic intervention of CYP1A1 could markedly attenuate colistin-induced cytotoxicity and apoptosis. Our current findings also provide insights into the underlying colistin-caused cytotoxicity.

## Data Availability

The data are contained within the article and Supplementary Materials.

## Supplementary Materials

The following supporting information can be downloaded at: https://www.mdpi.com/article/10.3390/antiox13070827/s1. Supplementary Materials and Methods: 2.10 sections. Supplementary Figure S1: The expression of CYP1A1 mRNA in PC12 cells transfected with SiCYP1A1#1 and #2. References [49,50] are cited in Supplementary Materials.
