# Supplementary material for: Colistin Induces Oxidative Stress and Apoptotic Cell Death through the Activation of the AhR/CYP1A1 Pathway in PC12 Cells
Source: Antioxidants (Basel). 2024 Jul 10;13(7):827. doi: 10.3390/antiox13070827 (PMC11273690; doi:10.3390/antiox13070827)
Supplement: Supplementary file 1 [file antioxidants-13-00827-s001.zip › antioxidants-3051411-supplementary.pdf]

Suppl. Materials and methods

2.10 Quantitative RT–PCR

The total RNAs of cell samples were extracted by a RNA Isolation Kit (Vazyme Biotech Co., Ltd., Nanjing, China) and the protocols were followed to a previous study [1]. In brief, the quality of isolated RNA was evaluated by the optical density at 260/280 nm (all values are between 1.9~2.1). Approximately 1 μg of total RNA was subjected to reverse transcription to produce cDNA by using the Prime Script RT-PCR kit (Takara, Dalian, China) and protocols are consistent with the manufacturer's instructions. The detail primer information is followed to the previous study [2], as below: β-actin (accession number: NM_031144.3) Forward 5′-AAC CCT AAG GCC AAC CGT GAA AAG-3′; β-actin Reverse 5′-CGA CCA GAG GCA TAC AGG GAC AAC-3′; AhR (accession number: NM_001308255.1) Forward 5′-TCA CTG CGC AGA ATC CCA CAT CC-3′; AhR Reverse 5′-TCG CGT CCT TCT TCA TCC GTT AGC-3′; CYP1A1 (accession number: XM_006243150.4) Forward 5′-GTC CCG GAT GTG GCC CTT CTC AAA-3′; CYP1A1 Reverse 5′-TAA CTC TTC CCT GGA TGC CTT CAA-3′; qRT-PCR were carried out by using a real-time PCR machine (AB7500 instrument, Applied Biosystems, USA). The conditions for all primer sets used were 95 °C for 20 sec., 55 °C for 30 sec., 72 °C for 30 sec. (45 cycles). β-actin was used as an internal control, and $2^{-\Delta\Delta Ct}$ method was used to calculate the fold change of gene expression.

References:

1. Dai, C.; Zhang, Q.; Shen, L.; Sharma, G.; Jiang, H.; Wang, Z.; Shen, J. Quercetin Attenuates Quinocetone-Induced Cell Apoptosis In Vitro by Activating the P38/Nrf2/HO-1 Pathway and Inhibiting the ROS/Mitochondrial Apoptotic Pathway. Antioxidants (Basel) 2022, 11, doi:10.3390/antiox11081498.
2. Moran, T.B.; Brannick, K.E.; Raetzman, L.T. Aryl-hydrocarbon receptor activity modulates prolactin expression in the pituitary. Toxicology and applied pharmacology 2012, 265, 139-145, doi:10.1016/j.taap.2012.08.026.

Supple. Figure S1

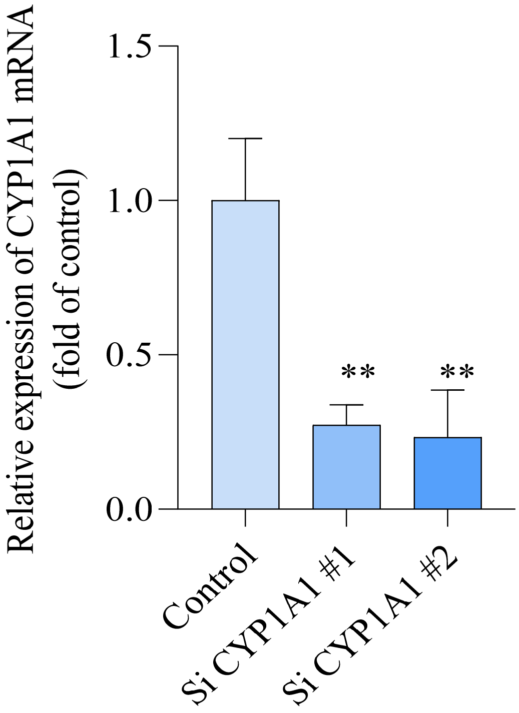

Supple. Figure S1 The expression of CYP1A1 mRNA in PC12 cells transfected with SiCYP1A1#1 and #2. The data is presented as mean ± SD (n = 3), with ** indicating significance at $P < 0.01$ compared to the untreated control group.
